# Systems Biology Approaches to Understand Natural Products Biosynthesis

**DOI:** 10.3389/fbioe.2015.00199

**Published:** 2015-12-09

**Authors:** Cuauhtemoc Licona-Cassani, Pablo Cruz-Morales, Angel Manteca, Francisco Barona-Gomez, Lars K. Nielsen, Esteban Marcellin

**Affiliations:** ^1^Australian Institute for Bioengineering and Nanotechnology (AIBN), The University of Queensland, Brisbane, QLD, Australia; ^2^National Laboratory of Genomics for Biodiversity (LANGEBIO), Centro de Investigación y de Estudios Avanzados del Instituto Politécnico Nacional (Cinvestav-IPN), Irapuato, México; ^3^Departamento de Biología Funcional and Instituto Universitario de Oncología del Principado de Asturias (IUOPA), Facultad de Medicina, Universidad de Oviedo, Oviedo, Spain

**Keywords:** actinomycetes, genome mining, genomics, transcriptomics, proteomics, metabolomics, genome-scale metabolic reconstructions

## Abstract

Actinomycetes populate soils and aquatic sediments that impose biotic and abiotic challenges for their survival. As a result, actinomycetes metabolism and genomes have evolved to produce an overwhelming diversity of specialized molecules. Polyketides, non-ribosomal peptides, post-translationally modified peptides, lactams, and terpenes are well-known bioactive natural products with enormous industrial potential. Accessing such biological diversity has proven difficult due to the complex regulation of cellular metabolism in actinomycetes and to the sparse knowledge of their physiology. The past decade, however, has seen the development of *omics* technologies that have significantly contributed to our better understanding of their biology. Key observations have contributed toward a shift in the exploitation of actinomycete’s biology, such as using their full genomic potential, activating entire pathways through key metabolic elicitors and pathway engineering to improve biosynthesis. Here, we review recent efforts devoted to achieving enhanced discovery, activation, and manipulation of natural product biosynthetic pathways in model actinomycetes using genome-scale biological datasets.

## Introduction

Actinomycetes represent one of the largest bacterial phyla and are primary contributors to carbon cycling and a major source of bioactive natural products (BNP) including, most prominently, antibiotics. Despite their prime importance, our understanding of actinomycete’s biology remains elusive owing to a characteristically large, convoluted, high GC content genome (Demain, 2014). The complexity of actinomycetes genomes was only fully revealed in the last decade as part of the genomic revolution. Sequencing of the first actinomycetes genomes revealed a plethora of bioactive secondary metabolites yet to be discovered in addition to the well-characterized biosynthetic gene clusters (BGC) (Doroghazi et al., 2014). According to NCBI database, to date around 1,000 actinomycete genomes have been fully sequenced and annotated. Homology sequence-based bioinformatic tools have confirmed their great potential as BNP producers; for example, species of *Streptomyces*, *Salinispora*, and *Saccharopolyspora* families contain an average of 30 secondary metabolite gene clusters (Nett et al., 2009).

**Figure 1:**
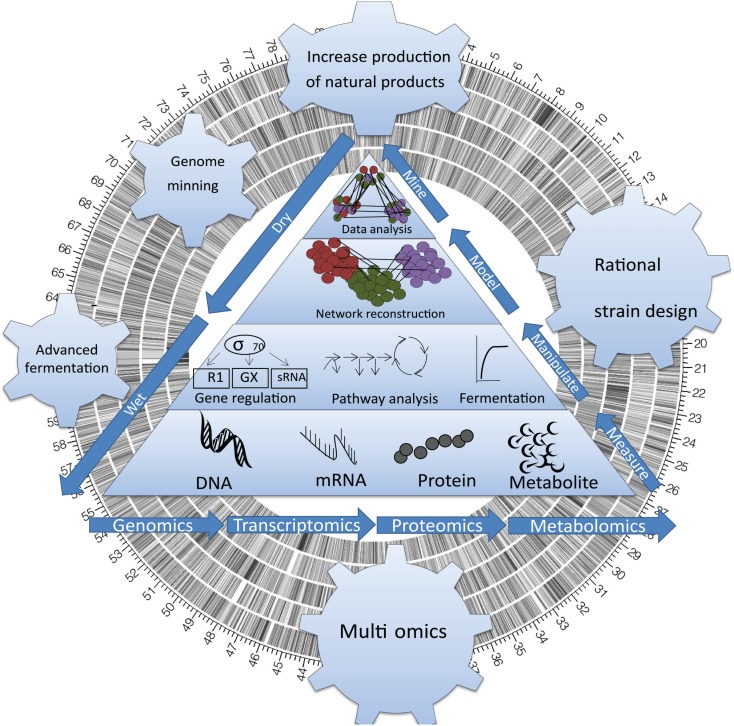
**Sequencing actinomycete genomes has revealed an unexpected complexity**. Systems Biology has opened access to the untapped chemical diversity encoded within the global microbial genome, including the vast majority (>99%) of taxa that are currently deemed unculturable, and a wealth of bioactive genes that are currently silent (untranslated) under standard cultivation condition.

The physiological changes leading to BNP biosynthesis in actinomycetes have been thoroughly studied over the past 10 years. Considerable work has advanced our understanding of the transitional stage triggering BNP biosynthesis (also known as the “metabolic switch”; Alam et al., 2010) and with it, our understanding of the physiological changes leading to secondary pathways activation. However, a lack of full understanding of this physiological transition stage has prevented us from manipulating fully this cellular process using metabolic engineering. Here, we review landmark studies contributing to the discovery, activation, and manipulation of metabolic pathways for BNP through the development of genome-wide biological datasets and systems biology in actinomycetes (Figure 1).

## Pathway Discovery: From Improved Genome Annotation to the Discovery of New BNP Biosynthetic Pathways

Genome annotation is the basis for pathway discovery and manipulation. Pathway discovery typically follows a defined pipeline: genome sequencing, annotation, gene discovery, and pathway manipulation. The exponential increase in sequencing efficiency is yielding an ever-increasing number of sequenced genomes, causing a bottleneck due to an often limited understanding of genome sequences. *In silico* approaches mainly rely on sequence homology scores to experimentally characterized sequences. Historically, however, functional microbiology has focused on a handful of microorganisms. Therefore, the genomic space for *in silico* genome annotation pipelines is biased for certain G + C content sequences, gene length, and organization. For instance, approximately 60% of the bacterial genomic space is miss-annotated in terms of gene boundaries (start/stop codons) caused by minimal cross-checks between computationally assigned open-reading frames (ORFs) and real genes (Nielsen and Krogh, 2005).

Bioinformatics-based pipelines failed to annotate accurately short-length proteins and high G + C content sequences in an annotation effort for 46 bacterial and archaea genomes (Venter et al., 2011). By contrast, functional annotations supported by “*omics* technologies” dramatically improve gene function assignment, particularly in less characterized microorganisms such as *Geobacter sulfurreducens* (Qiu et al., 2010) or the erythromycin producer actinomycete *Saccharopolyspora erythraea* (Marcellin et al., 2013a). Integration of proteomics and transcriptomics approaches has led to the re-annotation of these genomes, allowing for correction of hundreds of gene boundaries, the confirmation of hypothetical proteins and the discovery of dozens of new genes. A combination of proteomics and genomics, also known as proteogenomics, has also been used to deliver unbiased correlations between genome sequence and protein expression (Gupta et al., 2007; Gallien et al., 2009; Armengaud, 2010; Castellana and Bafna, 2010; Marcellin et al., 2013a).

### Genome Mining and Pathway Discovery

The first sequenced genomes of BNP producers were *Streptomyces coelicolor* (Bentley et al., 2002), *Streptomyces avermitilis* (Ikeda et al., 2003), producer of the insecticide/anthelmintic avermectin, *S. erythraea* (Oliynyk et al., 2007) and producer of the classic antibiotic streptomycin *Streptomyces griseus* (Ohnishi et al., 2008). Further sequence inspection of such genomes and other model actinomycetes have opened a plethora of BGCs, and revealed the great potential of actinomycetes genomes for the production of BNPs (Nett et al., 2009; Aigle et al., 2014; Doroghazi et al., 2014; Ikeda et al., 2014). However, exploiting this rich source of BNP has proven challenging. Genomic analyses show an abundance of known BGCs (i.e., chemically and genetically known), hypothetical BCGs (i.e., chemically unknown – genetically known), and cryptic BCGs (i.e., chemically unknown – genetically unknown) (Zerikly and Challis, 2009; Doroghazi and Metcalf, 2013).

Initial approaches to the discovery and identification of BNP were based on the search for cryptic BGC. The most common method involves gene mapping of enzymatic assembly complexes such as polyketide synthases (PKSs), non-ribosomal peptide synthases (NRPSs), and other enzymes typically related to BNPs (e.g., lanthipeptide synthases, terpene synthases, etc.). While simplistic, accumulation of structural, mechanistic, genetic, and chemical information on PKs and NRPs has allowed for the prediction of structures and chemical properties of dozens of BCGs from DNA sequences (Walsh et al., 2006; Hertweck, 2009; Jenke-Kodama and Dittmann, 2009; Koglin and Walsh, 2009; Walsh and Fischbach, 2010). Incorporating these mining strategies in specialized bioinformatic pipelines has revolutionized the genome mining scene efficiency. Genome-scale prediction of putative BGCs is nowadays possible within a few of hours.

Continuous progress has enabled the emergence of bioinformatics platforms, such as CLUSEAN (Weber et al., 2009), ClustScan (Starcevic et al., 2008), np.searcher (Li et al., 2009), SMURF (Khaldi et al., 2010), and antiSMASH (Medema et al., 2011b; Blin et al., 2013; Weber et al., 2015). The latter is the most popular system for automated BNP genome mining since it analyzes BGC domains to propose *loci*, chemical scaffold, and putative chemical structures. However, one of the biggest disadvantages of the use of these genome mining approaches is their intrinsic limitations to BGCs from known chemical structures. Complementary approaches have emerged to enable the discovery of novel BCGs, such as ClusterFinder (Cimermancic et al., 2014) or EvoMining (Medema and Fischbach, 2015). ClusterFinder uses hidden Markov model-based algorithms and Pfam as search database to annotate BCGs by clusters of protein domains with a biosynthetic logic. The use of ClusterFinder has allowed the detection of previously unknown classes of BCGs (Cimermancic et al., 2014). On the other hand, EvoMining is a functional phylogenomic pipeline that identifies expanded, repurposed enzyme families, with the potential to catalyze new conversions within BGC (Medema and Fischbach, 2015). This innovative method embraces the predictive power of evolutionary theory leading to model-independent predictions that include gene clusters that do not follow traditional biosynthetic rules. The method has been used for the discovery of the genes directing synthesis of small peptide aldehydes and the first biosynthetic system for arseno-organic metabolites.

Overall, genomic approaches have significantly improved the prediction of BNP from unannotated sequences and provided deep insights into the identification of novel chemical species. The genomic approach is limited to the known repertoire of BCGs, ignoring regulatory information for pathway activation.

## Pathway Activation: Systems Biology Analysis of Actinomycete Physiology and Development

Systems biology protocols have been successfully used to describe germination (Piette et al., 2005; Yagüe et al., 2013a; Bobek et al., 2014), programed cell death (Manteca et al., 2005), diauxic lag phase (Novotna et al., 2003), mutant analyses (bald A mutant) (Kim et al., 2005; Hesketh et al., 2007), and phosphate limitation (Rodríguez-García et al., 2007). Given that biosynthesis of natural products in actinomycetes is conceived as a physiological response to environmental changes (e.g., change of temperature, nutritional conditions, etc.), it is assumed that understanding their physiological behavior would provide the lead for natural product pathway activation and manipulation. Here, we focus on reviewing efforts devoted to understand the physiological transitions prior the activation of known natural product biosynthesis and the approaches used for the activation of unknown natural products biosynthetic pathways in model actinomycetes.

### Physiological Transitions and Development

Actinomycetes undergo drastic physiological changes during their developmental cycle (i.e., programed cell death and sporulation). In contrast to previous assumptions that sporulation events exclusively occurred in solid cultures (Flardh and Buttner, 2009), differentiation during pre-sporulation stages have been described in both solid and liquid *Streptomyces* cultures (Manteca et al., 2010). The existence of two different mycelia (MI and MII) across the developmental cycle has been characterized using iTRAQ LC-MS/MS proteomics, phosphoproteomics, and microarray-based transcriptomics (Manteca et al., 2010, 2011; Yagüe et al., 2013b). Specifically, proteins involved in antibiotic biosynthesis were upregulated in MII, and primary metabolism proteins from glycolysis, protein biosynthesis, and tricarboxylic acid cycle were upregulated in the MI. The second multinucleated mycelium with (aerial) and without (substrate) hydrophobic covers constituted a unique reproductive structure (Manteca et al., 2010). The most remarkable differences between MII from solid and liquid cultures involved proteins regulating the final stages of hyphae compartmentalization and spore formation (Manteca et al., 2010).

**Figure 2:**
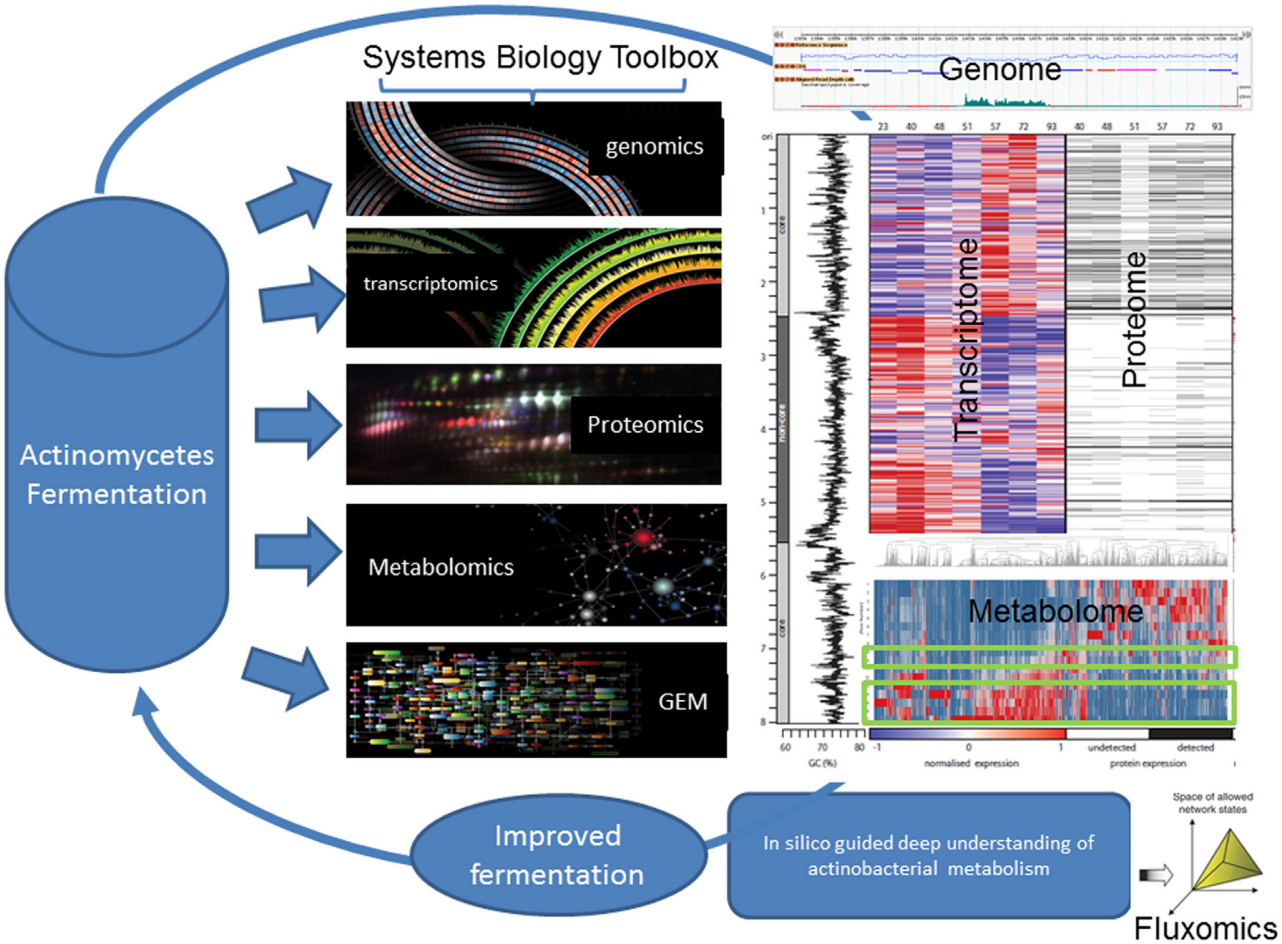
**Systems biology aims at understanding the larger picture of actinomycete’s biology – at the level of the organism – by putting its pieces together rather than apart**. It is in stark contrast to decades of reductionist biology in the area of actinomycete’s biology. For example, this figure illustrates the comprehensive multi-omics characterization of the *S. erythraea* metabolism across a fermentation time-course. As in most actinomycetes, at the 50th hour, there is a characteristic metabolic transition, which dictates the production of erythromycin. This figure illustrates how this transition is characterized by a massive loss of proteins and ribosomal RNA before a new expression pattern emerges. Around 30% of all transcripts arose from previously unannotated DNA and detailed analysis revealed approximately 350 new coding genes and 300 non-coding genes. Systems biology can unravel the complex nature of actinomycete’s biology at the transcriptomics, metabolomics, and proteomics level revealing various novel non-coding RNAs and uncharacterized phosphorylation patterns.

Similarly, characterization of the *S. erythraea* developmental cycle in bioreactors has been explored at base resolution transcription (RNA-seq), proteome (iTRAQ) and phosphoproteome (sMRM) (Marcellin et al., 2013a,b; Licona-Cassani et al., 2014) (Figure 2). The studies focused on the metabolic switch, a distinct transformational event that bisects two growth phases in actinomycetes and is characterized by rapid molecular and morphological changes. Authors found that the *S. erythraea* transcriptome undergoes extensive events of targeted mRNA degradation and transcription of mRNAs for adaptive metabolic functions, thereby resetting cells for the induction of a replacement transcriptional program. A suite of RNase and proteases mediate a targeted destruction of the transcriptome and proteome (suicidal patterns) in concert with the shifting of broad transcription macro-domains, delineated by core/non-core genomic regions. In addition, the temporal-dynamic, semi-quantitative phosphoproteomic study revealed that proteins from central metabolism (putative acetyl-CoA carboxylase, isocitrate lyase, and 2-oxoglutarate dehydrogenase) and key developmental pathways (trypsin-like serine protease, ribonuclease Rne/Rng, and ribosomal proteins) in *S. erythraea* change dramatically the degree of phosphorylation across the developmental cycle in liquid cultures (Figure 2) (Licona-Cassani et al., 2014).

One of the most significant observations linking actinomycete physiological behavior and pathway activation was made by Nieselt and collaborators (Nieselt et al., 2010). Using a temporal-dynamic transcriptomic analysis, Nieselt and collaborators identified the existence of several transitional stages along the fermentation that coincide with activation of natural product metabolic pathways in *S. coelicolor* (Nieselt et al., 2010). Under their bioreactor settings, early coordinated gene expression changes of genes related to nitrogen metabolism, including glutamine synthases I and II and the signaling protein GlnK is observed under similar temporal space as genes from the CPK antibiotic biosynthetic pathway. Interestingly, such transcriptional changes were observed under nitrogen sufficiency conditions. In addition, an unexpected transcriptional switch for developmental genes, such as chaplins, bldN, and whiH was registered showing for the first time that developmental genes are transcribed in *S. coelicolor* liquid cultures. Finally, the traditional metabolic switch was observed by a strong upregulation of the *pho* regulon together with the upregulation of the pigmented antibiotic undecylprodigiosin and actinorhodin (Nieselt et al., 2010).

While we are still far from overcoming the physiological barrier of achieving pathway activation and exploiting the full genomic potential of actinomycetes, systems biology approaches have significantly contributed to shifting key paradigms. First, we know that pathway activation does not follow the same regulatory rules to model actinomycetes (e.g., *S. coelicolor* or *Mycobacterium tuberculosis*); in fact, it is now possible to understand such differences in a single experiment. More importantly, systems biology has exposed a subset of strain-metabolite-specific regulatory mechanisms such as non-coding RNAs (Marcellin et al., 2013b), dynamic phosphorylation of ribosomal proteins (Licona-Cassani et al., 2014), acetylation of RNA degradation-related proteins (Huang et al., 2015), among others. The last part of this mini, review focuses on the efforts to manipulate metabolic pathways in actinomycetes using genome-scale metabolic reconstructions and metabolic engineering strategies.

## Pathway Manipulation: From Rational Design to Genome-Scale Model – Guided Metabolic Engineering Strategies in Actinomycetes

Production processes with sub-hundreds of mg/L product titers are considered unsustainable for industrial scale production. Actinomycetes cultures are also slow growing and unpredictable even under controlled conditions (i.e., bioreactor fermentations), and as such are difficult to ferment. For over 50 years, entire teams of metabolic and bioprocess engineers have used classical approaches such as random mutagenesis rounds (Tanaka et al., 2009; Jung et al., 2011), media design, and process optimization (Hamedi et al., 2004; El-Enshasy et al., 2008; Zou et al., 2009), and rationally designed metabolic engineering strategies (Reeves et al., 2006, 2007; Olano et al., 2008) to engineer actinomycetes bioprocesses and strains to achieve acceptable titers. Modern strain engineering uses genome-scale models (GEMs) in combination with *omics* data for the integration of genome-scale biological datasets toward the manipulation of metabolism.

Since the first GEM release, more than a decade ago (Edwards and Palsson, 1999), applications have expanded from *in silico* metabolic predictions (Edwards and Palsson, 2000; Schilling et al., 2002; Park et al., 2007) to the discovery of antibacterial targets (Oberhardt et al., 2009; Kim et al., 2010), integration of biological datasets (Lerman et al., 2012; Imam et al., 2015), phenotype prediction (O’Brien et al., 2013), estimation of metabolic capabilities (O’Brien et al., 2015), and the study of evolutionary relationships of metabolic and regulatory networks (Oberhardt et al., 2009; Kim et al., 2010; Barona-Gomez et al., 2012).

While draft GEM are now routinely generated using high-throughput automated pipelines (Aziz et al., 2008; Henry et al., 2010), successful applications of GEMs to microbial metabolism only have occurred on exhaustively (manually) curated/experimentally validated metabolic reconstructions. In this regard, despite being our primary microbial source for antibiotics, only a handful of manually curated GEM for actinomycetes have been reported (Borodina et al., 2005a; Beste et al., 2007; Jamshidi and Palsson, 2007; Alam et al., 2010; Medema et al., 2010, 2011a; Chindelevitch et al., 2012; Licona-Cassani et al., 2012). Even more surprising is the fact that pathway optimization of actinomycete metabolism has only been achieved at the level of precursor supply (Borodina et al., 2005b, 2008; Licona-Cassani et al., 2012). In such approaches, optimal solutions are found because there is congruence between cellular (maximize growth rate) and engineering objectives (maximize productivity). In order to properly optimize production of non-growth-associated metabolites (i.e., BNP) novel algorithms and new objective functions are to be incorporated to current protocols.

The last few years have seen the emergence of network reconstruction beyond metabolism. These next-generation network reconstructions account for expression coupled to metabolism and even transcriptional regulation. The first such model, known as ME-model, was developed for *Thermotoga maritima* (Zhang et al., 2009; Lerman et al., 2012) rapidly followed by the *Escherichia coli* ME-model (O’Brien et al., 2013). Incorporation of gene expression in the mathematical framework allows these models to expand their predictive capabilities, which may be what is needed to model non-growth-associated metabolites such as BNPs. It is expected that as ME-models for actinomycetes become available, just like GEMs became available 10 years ago, multi-omics integration may be possible, and with it, models become more predictive.

## Future Directions

Like in model organisms, such as yeast and *E. coli*, systems biology in actinomycetes has immensely advanced our understanding of this complex and fascinating bacterial family, offering insightful information regarding gene discovery, gene regulation, and pathway manipulation. The tools are highly developed and readily available yet integration and data analysis remain our main challenge. Just like finding a needle in a haystack, finding a key gene in a sea of data is extremely challenging; the main challenge remains the lack of tools for integration and visualization of large datasets. As such, systems biology in actinomycetes has yet to deliver real advances for the production of BNPs and discovery of novel bioactive compounds.

## Conflict of Interest Statement

The authors declare that the research was conducted in the absence of any commercial or financial relationships that could be construed as a potential conflict of interest.

## References

[B1] Aigle B. Lautru S. Spiteller D. Dickschat J. Challis G. Leblond P. (2014). Genome mining of *Streptomyces ambofaciens*. J. Ind. Microbiol. Biotechnol. 41, 251–263. 10.1007/s10295-013-1379-y 24258629

[B2] Alam M. T. Merlo M. Consortium T. S. Hodgson D. Wellington E. Takano E. (2010). Metabolic modeling and analysis of the metabolic switch in *Streptomyces coelicolor*. BMC Genomics 11:202. 10.1186/1471-2164-11-202 20338070 PMC2853524

[B3] Armengaud J. (2010). Proteogenomics and systems biology: quest for the ultimate missing parts. Expert Rev. Proteomics 7, 65–77. 10.1586/Epr.09.104 20121477

[B4] Aziz R. Bartels D. Best A. Dejongh M. Disz T. Edwards R. (2008). The RAST server: rapid annotations using subsystems technology. BMC Genomics 9:75. 10.1186/1471-2164-9-75 18261238 PMC2265698

[B5] Barona-Gomez F. Cruz-Morales P. Noda-García L. (2012). What can genome-scale metabolic network reconstructions do for prokaryotic systematics? Antonie Van Leeuwenhoek 101, 35–43. 10.1007/s10482-011-9655-1 22016333

[B6] Bentley S. Chater K. Cerdeno-Tarraga A.-M. Challis G. Thomson N. James K. (2002). Complete genome sequence of the model actinomycete *Streptomyces coelicolor* A3(2). Nature 417, 141–147. 10.1038/417141a 12000953

[B7] Beste D. Hooper T. Stewart G. Bonde B. Avignone-Rossa C. Bushell M. (2007). GSMN-TB: a web-based genome-scale network model of *Mycobacterium tuberculosis* metabolism. Genome Biol. 8, R89. 10.1186/gb-2007-8-5-r89 17521419 PMC1929162

[B8] Blin K. Medema M. H. Kazempour D. Fischbach M. A. Breitling R. Takano E. (2013). antiSMASH 2.0-a versatile platform for genome mining of secondary metabolite producers. Nucleic Acids Res. 41, W204–W212. 10.1093/nar/gkt449 23737449 PMC3692088

[B9] Bobek J. Strakova E. Zikova A. Vohradsky J. (2014). Changes in activity of metabolic and regulatory pathways during germination of *S. coelicolor*. BMC Genomics 15:1173. 10.1186/1471-2164-15-1173 25539760 PMC4367926

[B10] Borodina I. Krabben P. Nielsen J. (2005a). Genome-scale analysis of *Streptomyces coelicolor* A3(2) metabolism. Genome Res. 15, 820–829. 10.1101/gr.3364705 15930493 PMC1142472

[B11] Borodina I. Schöller C. Eliasson A. Nielsen J. (2005b). Metabolic network analysis of *Streptomyces tenebrarius*, a *Streptomyces* species with an active Entner-Doudoroff pathway. Appl. Environ. Microbiol. 71, 2294–2302. 10.1128/aem.71.5.2294-2302.2005 15870314 PMC1087532

[B12] Borodina I. Siebring J. Zhang J. Smith C. P. Van Keulen G. Dijkhuizen L. (2008). Antibiotic overproduction in *Streptomyces coelicolor* A3(2) mediated by phosphofructokinase deletion. J. Biol. Chem. 283, 25186–25199. 10.1074/jbc.M803105200 18606812

[B13] Castellana N. Bafna V. (2010). Proteogenomics to discover the full coding content of genomes: a computational perspective. J. Proteomics 73, 2124–2135. 10.1016/j.jprot.2010.06.007 20620248 PMC2949459

[B14] Chindelevitch L. Stanley S. Hung D. Regev A. Berger B. (2012). MetaMerge: scaling up genome-scale metabolic reconstructions with application to *Mycobacterium tuberculosis*. Genome Biol. 13, r6. 10.1186/gb-2012-13-1-r6 22292986 PMC3488975

[B15] Cimermancic P. Medema M. H. Claesen J. Kurita K. Wieland Brown L. C. Mavrommatis K. (2014). Insights into secondary metabolism from a global analysis of prokaryotic biosynthetic gene clusters. Cell 158, 412–421. 10.1016/j.cell.2014.06.034 25036635 PMC4123684

[B16] Demain A. (2014). Importance of microbial BNP and the need to revitalize their discovery. J. Ind. Microbiol. Biotechnol. 41, 185–201. 10.1007/s10295-013-1325-z 23990168

[B17] Doroghazi J. Metcalf W. (2013). Comparative genomics of actinomycetes with a focus on natural product biosynthetic genes. BMC Genomics 14:611. 10.1186/1471-2164-14-611 24020438 PMC3848822

[B18] Doroghazi J. R. Albright J. C. Goering A. W. Ju K.-S. Haines R. R. Tchalukov K. A. (2014). A roadmap for natural product discovery based on large-scale genomics and metabolomics. Nat. Chem. Biol. 10, 963–968. 10.1038/nchembio.1659 25262415 PMC4201863

[B19] Edwards J. S. Palsson B. O. (1999). Systems properties of the *Haemophilus influenzae* metabolic genotype. J. Biol. Chem. 274, 17410–17416. 10364169 10.1074/jbc.274.25.17410

[B20] Edwards J. S. Palsson B. O. (2000). The *Escherichia coli* MG1655 in silico metabolic genotype: its definition, characteristics, and capabilities. Proc. Natl. Acad. Sci. U.S.A. 97, 5528–5533. 10.1073/pnas.97.10.5528 10805808 PMC25862

[B21] El-Enshasy H. A. Mohamed N. A. Farid M. A. El-Diwany A. I. (2008). Improvement of erythromycin production by *Saccharopolyspora erythraea* in molasses based medium through cultivation medium optimization. Bioresour. Technol. 99, 4263–4268. 10.1016/j.biortech.2007.08.050 17936622

[B22] Flardh K. Buttner M. J. (2009). *Streptomyces* morphogenetics: dissecting differentiation in a filamentous bacterium. Nat. Rev. Microbiol. 7, 36–49. 10.1038/nrmicro1968 19079351

[B23] Gallien S. Perrodou E. Carapito C. Deshayes C. Reyrat J. M. Van Dorsselaer A. (2009). Ortho-proteogenomics: multiple proteomes investigation through orthology and a new MS-based protocol. Genome Res. 19, 128–135. 10.1101/gr.0s81901.108 18955433 PMC2612966

[B24] Gupta N. Tanner S. Jaitly N. Adkins J. N. Lipton M. Edwards R. (2007). Whole proteome analysis of post-translational modifications: applications of mass-spectrometry for proteogenomic annotation. Genome Res. 17, 1362–1377. 10.1101/Gr.6427907 17690205 PMC1950905

[B25] Hamedi J. Malekzadeh F. Saghafi-Nia A. E. (2004). Enhancing of erythromycin production by *Saccharopolyspora erythraea* with common and uncommon oils. J. Ind. Microbiol. Biotechnol. 31, 447–456. 10.1007/s10295-004-0166-1 15480942

[B26] Henry C. S. Dejongh M. Best A. A. Frybarger P. M. Linsay B. Stevens R. L. (2010). High-throughput generation, optimization and analysis of genome-scale metabolic models. Nat. Biotechnol. 28, 977–982. 10.1038/nbt.1672 20802497

[B27] Hertweck C. (2009). The biosynthetic logic of polyketide diversity. Angew. Chem. Int. Ed. 48, 4688–4716. 10.1002/anie.200806121 19514004

[B28] Hesketh A. Bucca G. Laing E. Flett F. Hotchkiss G. Smith C. (2007). New pleiotropic effects of eliminating a rare tRNA from *Streptomyces coelicolor*, revealed by combined proteomic and transcriptomic analysis of liquid cultures. BMC Genomics 8:261. 10.1186/1471-2164-8-261 17678549 PMC2000904

[B29] Huang D. Li Z.-H. You D. Zhou Y. Ye B.-C. (2015). Lysine acetylproteome analysis suggests its roles in primary and secondary metabolism in *Saccharopolyspora erythraea*. Appl. Microbiol. Biotechnol. 99, 1399–1413. 10.1007/s00253-014-6144-2 25487885

[B30] Ikeda H. Ishikawa J. Hanamoto A. Shinose M. Kikuchi H. Shiba T. (2003). Complete genome sequence and comparative analysis of the industrial microorganism *Streptomyces avermitilis*. Nat. Biotechnol. 21, 526–531. 10.1038/nbt820 12692562

[B31] Ikeda H. Shin-Ya K. Omura S. (2014). Genome mining of the *Streptomyces avermitilis* genome and development of genome-minimized hosts for heterologous expression of biosynthetic gene clusters. J. Ind. Microbiol. Biotechnol. 41, 233–250. 10.1007/s10295-013-1327-x 23990133

[B32] Imam S. Schäuble S. Brooks A. N. Baliga N. S. Price N. D. (2015). Data-driven integration of genome-scale regulatory and metabolic network models. Front. Microbiol. 6:409. 10.3389/fmicb.2015.00409 25999934 PMC4419725

[B33] Jamshidi N. Palsson B. (2007). Investigating the metabolic capabilities of *Mycobacterium tuberculosis* H37Rv using the in silico strain iNJ661 and proposing alternative drug targets. BMC Syst. Biol. 1:26. 10.1186/1752-0509-1-26 17555602 PMC1925256

[B34] Jenke-Kodama H. Dittmann E. (2009). Bioinformatic perspectives on NRPS/PKS megasynthases: advances and challenges. Nat. Prod. Rep. 26, 874–883. 10.1039/b810283j 19554239

[B35] Jung W. Yoo Y. Park J. Park S. Han A. Ban Y. (2011). A combined approach of classical mutagenesis and rational metabolic engineering improves rapamycin biosynthesis and provides insights into methylmalonyl-CoA precursor supply pathway in *Streptomyces hygroscopicus* ATCC 29253. Appl. Microbiol. Biotechnol. 91, 1389–1397. 10.1007/s00253-011-3348-6 21655985

[B36] Khaldi N. Seifuddin F. T. Turner G. Haft D. Nierman W. C. Wolfe K. H. (2010). SMURF: genomic mapping of fungal secondary metabolite clusters. Fungal Genet. Biol. 47, 736–741. 10.1016/j.fgb.2010.06.003 20554054 PMC2916752

[B37] Kim D.-W. Chater K. Lee K.-J. Hesketh A. (2005). Changes in the extracellular proteome caused by the absence of the bldA gene product, a developmentally significant tRNA, reveal a new target for the pleiotropic regulator adpA in *Streptomyces coelicolor*. J. Bacteriol. 187, 2957–2966. 10.1128/jb.187.9.2957-2966.2005 15838021 PMC1082842

[B38] Kim T. Y. Kim H. U. Lee S. Y. (2010). Metabolite-centric approaches for the discovery of antibacterials using genome-scale metabolic networks. Metab. Eng. 12, 105–111. 10.1016/j.ymben.2009.05.004 19481614

[B39] Koglin A. Walsh C. T. (2009). Structural insights into nonribosomal peptide enzymatic assembly lines. Nat. Prod. Rep. 26, 987–1000. 10.1039/b904543k 19636447 PMC2773127

[B40] Lerman J. A. Hyduke D. R. Latif H. Portnoy V. A. Lewis N. E. Orth J. D. (2012). In silico method for modelling metabolism and gene product expression at genome scale. Nat. Commun. 3, 929. 10.1038/ncomms1928 22760628 PMC3827721

[B41] Li M. H. Ung P. M. Zajkowski J. Garneau-Tsodikova S. Sherman D. H. (2009). Automated genome mining for natural products. BMC Bioinformatics 10:185. 10.1186/1471-2105-10-185 19531248 PMC2712472

[B42] Licona-Cassani C. Lim S. Marcellin E. Nielsen L. K. (2014). Temporal dynamics of the *Saccharopolyspora erythraea* phosphoproteome. Mol. Cell. Proteomics 13, 1219–1230. 10.1074/mcp.M113.033951 24615062 PMC4014280

[B43] Licona-Cassani C. Marcellin E. Quek L.-E. Jacob S. Nielsen L. (2012). Reconstruction of the *Saccharopolyspora erythraea* genome-scale model and its use for enhancing erythromycin production. Antonie Van Leeuwenhoek 102, 493–502. 10.1007/s10482-012-9783-2 22847261

[B44] Manteca A. Fernandez M. Sanchez J. (2005). Mycelium development in *Streptomyces antibioticus* ATCC11891 occurs in an orderly pattern which determines multiphase growth curves. BMC Microbiol. 5:51. 10.1186/1471-2180-5-51 16164744 PMC1249576

[B45] Manteca A. Jung H. R. Schwa¨mmle V. Jensen O. N. Sanchez J. (2010). Quantitative proteome analysis of *Streptomyces coelicolor* nonsporulating liquid cultures demonstrates a complex differentiation process comparable to that occurring in sporulating solid cultures. J. Proteome Res. 9, 4801–4811. 10.1021/pr100513p 20681593

[B46] Manteca A. Ye J. Sanchez J. Jensen O. N. (2011). Phosphoproteome analysis of *Streptomyces* development reveals extensive protein phosphorylation accompanying bacterial differentiation. J. Proteome Res. 10, 5481–5492. 10.1021/pr200762y 21999169

[B47] Marcellin E. Licona-Cassani C. Mercer T. Palfreyman R. Nielsen L. (2013a). Re-annotation of the *Saccharopolyspora erythraea* genome using a systems biology approach. BMC Genomics 14:699 10.1186/1471-2164-14-699 24118942 PMC4008361

[B48] Marcellin E. Mercer T. R. Licona-Cassani C. Palfreyman R. W. Dinger M. E. Steen J. A. (2013b). *Saccharopolyspora erythraea*’s genome is organised in high-order transcriptional regions mediated by targeted degradation at the metabolic switch. BMC Genomics 14:15 10.1186/1471-2164-14-15 23324121 PMC3610266

[B49] Medema M. H. Alam M. T. Heijne W. H. M. Van Den Berg M. A. Müller U. Trefzer A. (2011a). Genome-wide gene expression changes in an industrial clavulanic acid overproduction strain of *Streptomyces clavuligerus*. Microb. Biotechnol. 4, 300–305. 10.1111/j.1751-7915.2010.00226.x 21342474 PMC3818869

[B50] Medema M. H. Blin K. Cimermancic P. De Jager V. Zakrzewski P. Fischbach M. A. (2011b). antiSMASH: rapid identification, annotation and analysis of secondary metabolite biosynthesis gene clusters in bacterial and fungal genome sequences. Nucleic Acids Res. 39, W339–W346. 10.1093/nar/gkr466 21672958 PMC3125804

[B51] Medema M. H. Fischbach M. A. (2015). Computational approaches of natural product discovery. Nat. Chem. Biol. 11, 639–648. 10.1038/nchembio.1884 26284671 PMC5024737

[B52] Medema M. H. Trefzer A. Kovalchuk A. Van Den Berg M. Muller U. Heijne W. (2010). The sequence of a 1.8-Mb bacterial linear plasmid reveals a rich evolutionary reservoir of secondary metabolic pathways. Genome Biol. Evol. 2, 212–224. 10.1093/gbe/evq013 20624727 PMC2997539

[B53] Nett M. Ikeda H. Moore B. S. (2009). Genomic basis for natural product biosynthetic diversity in the actinomycetes. Nat. Prod. Rep. 26, 1362–1384. 10.1039/b817069j 19844637 PMC3063060

[B54] Nielsen P. Krogh A. (2005). Large-scale prokaryotic gene prediction and comparison to genome annotation. Bioinformatics 21, 4322–4329. 10.1093/bioinformatics/bti701 16249266

[B55] Nieselt K. Battke F. Herbig A. Bruheim P. Wentzel A. Jakobsen O. (2010). The dynamic architecture of the metabolic switch in *Streptomyces coelicolor*. BMC Genomics 11:10. 10.1186/1471-2164-11-10 20053288 PMC2824715

[B56] Novotna J. Vohradsky J. Berndt P. Gramajo H. Langen H. Li X.-M. (2003). Proteomic studies of diauxic lag in the differentiating prokaryote *Streptomyces coelicolor* reveal a regulatory network of stress-induced proteins and central metabolic enzymes. Mol. Microbiol. 48, 1289–1303. 10.1046/j.1365-2958.2003.03529.x 12787356

[B57] Oberhardt M. A. Palsson B. O. Papin J. A. (2009). Applications of genome-scale metabolic reconstructions. Mol. Syst. Biol. 5, 320. 10.1038/msb.2009.77 19888215 PMC2795471

[B58] O’Brien E. J. Lerman J. A. Chang R. L. Hyduke D. R. Palsson B. Ø (2013). Genome-scale models of metabolism and gene expression extend and refine growth phenotype prediction. Mol. Syst. Biol. 9, 693. 10.1038/msb.2013.52 24084808 PMC3817402

[B59] O’Brien E. J. Monk J. M. Palsson B. O. (2015). Using genome-scale models to predict biological capabilities. Cell 161, 971–987. 10.1016/j.cell.2015.05.019 26000478 PMC4451052

[B60] Ohnishi Y. Ishikawa J. Hara H. Suzuki H. Ikenoya M. Ikeda H. (2008). Genome sequence of the streptomycin-producing microorganism *Streptomyces griseus* IFO 13350. J. Bacteriol. 190, 4050–4060. 10.1128/jb.00204-08 18375553 PMC2395044

[B61] Olano C. Lombó F. Méndez C. Salas J. A. (2008). Improving production of bioactive secondary metabolites in actinomycetes by metabolic engineering. Metab. Eng. 10, 281–292. 10.1016/j.ymben.2008.07.001 18674632

[B62] Oliynyk M. Samborskyy M. Lester J. B. Mironenko T. Scott N. Dickens S. (2007). Complete genome sequence of the erythromycin-producing bacterium *Saccharopolyspora erythraea* NRRL23338. Nat. Biotechnol. 25, 447–453. 10.1038/nbt1297 17369815

[B63] Park J. H. Lee K. H. Kim T. Y. Lee S. Y. (2007). Metabolic engineering of *Escherichia coli* for the production of L-valine based on transcriptome analysis and in silico gene knockout simulation. Proc. Natl. Acad. Sci. U.S.A. 104, 7797–7802. 10.1073/pnas.0702609104 17463081 PMC1857225

[B64] Piette A. Derouaux A. Gerkens P. Noens E. E. E. Mazzucchelli G. Vion S. (2005). From dormant to germinating spores of *Streptomyces coelicolor* A3(2): New perspectives from the crp null mutant. J. Proteome Res. 4, 1699–1708. 10.1021/pr050155b 16212423

[B65] Qiu Y. Cho B.-K. Park Y. S. Lovley D. Palsson B. O. Zengler K. (2010). Structural and operational complexity of the *Geobacter sulfurreducens* genome. Genome Res. 20, 1304–1311. 10.1101/gr.107540.110 20592237 PMC2928509

[B66] Reeves A. Brikun I. Cernota W. Leach B. Gonzalez M. Weber J. (2006). Effects of methylmalonyl-CoA mutase gene knockouts on erythromycin production in carbohydrate-based and oil-based fermentations of *Saccharopolyspora erythraea*. J. Ind. Microbiol. Biotechnol. 33, 600–609. 10.1007/s10295-006-0094-3 16491356

[B67] Reeves A. R. Brikun I. A. Cernota W. H. Leach B. I. Gonzalez M. C. Mark Weber J. (2007). Engineering of the methylmalonyl-CoA metabolite node of *Saccharopolyspora erythraea* for increased erythromycin production. Metab. Eng. 9, 293–303. 10.1016/j.ymben.2007.02.001 17482861 PMC2722834

[B68] Rodríguez-García A. Barreiro C. Santos-Beneit F. Sola-Landa A. Martín J. F. (2007). Genome-wide transcriptomic and proteomic analysis of the primary response to phosphate limitation in *Streptomyces coelicolor* M145 and in a ∆*phoP* mutant. Proteomics 7, 2410–2429. 10.1002/pmic.200600883 17623301

[B69] Schilling C. H. Covert M. W. Famili I. Church G. M. Edwards J. S. Palsson B. O. (2002). Genome-scale metabolic model of *Helicobacter pylori* 26695. J. Bacteriol. 184, 4582–4593. 10.1128/jb.184.16.4582-4593.2002 12142428 PMC135230

[B70] Starcevic A. Zucko J. Simunkovic J. Long P. F. Cullum J. Hranueli D. (2008). ClustScan: an integrated program package for the semi-automatic annotation of modular biosynthetic gene clusters and in silico prediction of novel chemical structures. Nucleic Acids Res. 36, 6882–6892. 10.1093/nar/gkn685 18978015 PMC2588505

[B71] Tanaka Y. Komatsu M. Okamoto S. Tokuyama S. Kaji A. Ikeda H. (2009). Antibiotic overproduction by *rpsL* and *rsmG* mutants of various actinomycetes. Appl. Environ. Microbiol. 75, 4919–4922. 10.1128/AEM.00681-09 19447953 PMC2708438

[B72] Venter E. Smith R. D. Payne S. H. (2011). Proteogenomic analysis of bacteria and archaea: a 46 organism case study. PLoS ONE 6:e27587. 10.1371/journal.pone.0027587 22114679 PMC3219674

[B73] Walsh C. T. Fischbach M. A. (2010). BNP version 2.0: connecting genes to molecules. J. Am. Chem. Soc. 132, 2469–2493. 10.1021/ja909118a 20121095 PMC2828520

[B74] Walsh C. T. Garneau-Tsodikova S. Howard-Jones A. R. (2006). Biological formation of pyrroles: nature’s logic and enzymatic machinery. Nat. Prod. Rep. 23, 517–531. 10.1039/b605245m 16874387

[B75] Weber T. Blin K. Duddela S. Krug D. Kim H. U. Bruccoleri R. (2015). antiSMASH 3.0-a comprehensive resource for the genome mining of biosynthetic gene clusters. Nucleic Acids Res. 43, W237–W243. 10.1093/nar/gkv437 25948579 PMC4489286

[B76] Weber T. Rausch C. Lopez P. Hoof I. Gaykova V. Huson D. H. (2009). CLUSEAN: a computer-based framework for the automated analysis of bacterial secondary metabolite biosynthetic gene clusters. J. Biotechnol. 140, 13–17. 10.1016/j.jbiotec.2009.01.007 19297688

[B77] Yagüe P. López-García M. T. Rioseras B. Sánchez J. Manteca Á (2013a). Pre-sporulation stages of *Streptomyces* differentiation: state-of-the-art and future perspectives. FEMS Microbiol. Lett. 342, 79–88. 10.1111/1574-6968.12128 23496097 PMC3654496

[B78] Yagüe P. Rodríguez-García A. López-García M. T. Martín J. F. Rioseras B. Sánchez J. (2013b). Transcriptomic analysis of *Streptomyces coelicolor* differentiation in solid sporulating cultures: first compartmentalized and second multinucleated mycelia have different and distinctive transcriptomes. PLoS ONE 8:e60665 10.1371/journal.pone.0060665 23555999 PMC3610822

[B79] Zerikly M. Challis G. L. (2009). Strategies for the discovery of new BNP by genome mining. Chembiochem 10, 625–633. 10.1002/cbic.200800389 19165837

[B80] Zhang Y. Thiele I. Weekes D. Li Z. Jaroszewski L. Ginalski K. (2009). Three-dimensional structural view of the central metabolic network of *Thermotoga maritima*. Science 325, 1544–1549. 10.1126/science.1174671 19762644 PMC2833182

[B81] Zou X. Hang H.-F. Chu J. Zhuang Y.-P. Zhang S.-L. (2009). Oxygen uptake rate optimization with nitrogen regulation for erythromycin production and scale-up from 50 L to 372 m3 scale. Bioresour. Technol. 100, 1406–1412. 10.1016/j.biortech.2008.09.017 18929481

